# A novel use of AI for prediction of clinical deterioration in a post-acute hospital

**DOI:** 10.1038/s44401-026-00145-5

**Published:** 2026-09-02

**Authors:** Alexander Kauffman, Nataša Lazarević, Rahul Joshi, Jordan Pelc

**Affiliations:** 1 Division of Hospital Medicine, Sinai Health, Toronto, ON Canada; 2 Human Factors and Design, Sinai Health, Toronto, ON Canada; 3 Department of Family and Community Medicine, Temerty Faculty of Medicine, University of Toronto, Toronto, ON Canada

**Keywords:** Computational biology and bioinformatics, Diseases, Health care, Mathematics and computing, Medical research

## Abstract

There is a growing literature on the prediction of risk of deterioration in hospital settings, including by leveraging artificial intelligence (AI) models. However, this literature has focused on acute-care hospitals, rather than post-acute facilities, where the risk of deterioration remains high. Post-acute facilities tend to have lower digital maturity and poorer data foundations, as well as less rich physiologic data, making the implementation of AI tools for deterioration challenging. In this study, we demonstrate a novel use of AI for the prediction of clinical deterioration in a post-acute hospital. Despite clinical and technical barriers, we developed a meaningful predictive model (sensitivity = 82%). Further, the patterns of contribution to risk of deterioration from the primary contributing variables were remarkably physiologic. This demonstrates a novel model for the prediction of deterioration in an understudied context and provides novel evidence for physiologic predictors of deterioration consistent with previous acute-care scoring systems in the post-acute environment.

## Introduction

Identification of clinical deterioration in hospital settings is essential for patient safety^1,2^. Nearly all the work in this area has been done in acute-care environments, where there are established models for predicting deterioration based on common clinical parameters^3^. More advanced technologies are also used in the acute-care setting for predicting deterioration, namely artificial intelligence (AI) models^4,5^. AI models have shown superior performance in predicting deterioration in acute hospitals, leveraging high-frequency monitoring and continuous physiologic data streams. By contrast, post-acute care facilities have been the focus of far fewer studies in this area. Common models of patient deterioration have not been validated in the post-acute setting, and therefore, important predictors of clinical deterioration in this setting may not be known. More advanced predictive approaches are not typically technologically feasible in post-acute hospitals because they lack advanced monitoring systems. These facilities also have lower digital maturity, often relying on legacy EMR systems with poorer data foundations and analytic capabilities^6^.

A fundamental conceptual problem in predicting clinical deterioration in post-acute settings is *defining* clinical deterioration in these settings. Clinical deterioration is understood to mean that clinically, a patient requires an escalation of care, that is, an increased level of medical care beyond what they are currently receiving. In the acute hospital context, an increased level of care generally means an acute intervention to maintain physiologic viability, such as the interventions provided in critical-care settings. As such, in the acute setting, clinical deterioration represents physiologic deterioration. In the post-acute context, where hospitals are designed for rehabilitation and chronic care rather than acute illness, *any* acute clinical scenario beyond the resources of the medical and nursing staff to manage in-house may need an escalation of care, namely, transfer to an acute-care hospital. This can include all manner of acute illnesses, including potential physiologic crises such as sepsis or cardiac decompensation, but also falls with fractures requiring surgical intervention, unexplained mental status changes, and psychiatric disturbances, which are not necessarily associated with physiologic instability. Clinical deterioration in the post-acute hospital, therefore, encompasses a broad range of concerns that, though real clinical entities, are defined by a final common *workflow* pathway, namely, escalation to an acute-care facility, rather than similar physiologic features. This poses further predictability challenges given the heterogeneity of reasons for acute-care transfer and possible decreased utility of traditional variables such as vital signs for predicting deterioration when applied to some of these circumstances.

Despite these challenges, the risk of clinical deterioration is high, with some studies estimating a 30% rate of readmission to acute-care hospitals among patients admitted to post-acute facilities^7^. Our team has been working to improve the process of managing patient deterioration in the post-acute environment. Given the lack of predictive models available for use in this setting, we sought to develop a local approach based on aggregate patient data to predict deterioration in order to improve patient safety. Our team therefore worked to solve two problems concurrently: one, to develop a predictive model for clinical deterioration in a post-acute hospital; and two, to facilitate this, to develop an approach to the application of cutting-edge predictive techniques, namely, AI, to a digitally immature facility reliant on a legacy EMR.

To meet these objectives, we developed, trained, and evaluated six supervised machine learning models using structured EMR data from a post-acute hospital. We then assessed the best-performing model to identify the most influential predictors and evaluate whether the learned patterns were clinically plausible.

## Results

A total of 2711 patients were included in the final study cohort. Population demographics are described in Table 1. The population was predominantly older adults, with a clinical deterioration rate of 22.6%, indicating a substantially imbalanced outcome distribution suitable for machine learning-based risk prediction.

**Table 1:** Baseline demographics and clinical characteristics of the study population

Characteristic	Overall cohort
*N* (unique patients)	2711
Age, years (mean ± SD)	73.6 ± 14.7
Sex, *n* (%)—Female	1414 (52.2%)
Sex, *n* (%)—Male	1297 (47.8%)
Length of stay, days (mean ± SD)	42 ± 46.0
Deterioration events, *n* (%)	612 (22.6%)

Continuous variables are presented as mean ± standard deviation (SD). Categorical variables are presented as a number (percentage).

**Fig. 1:**
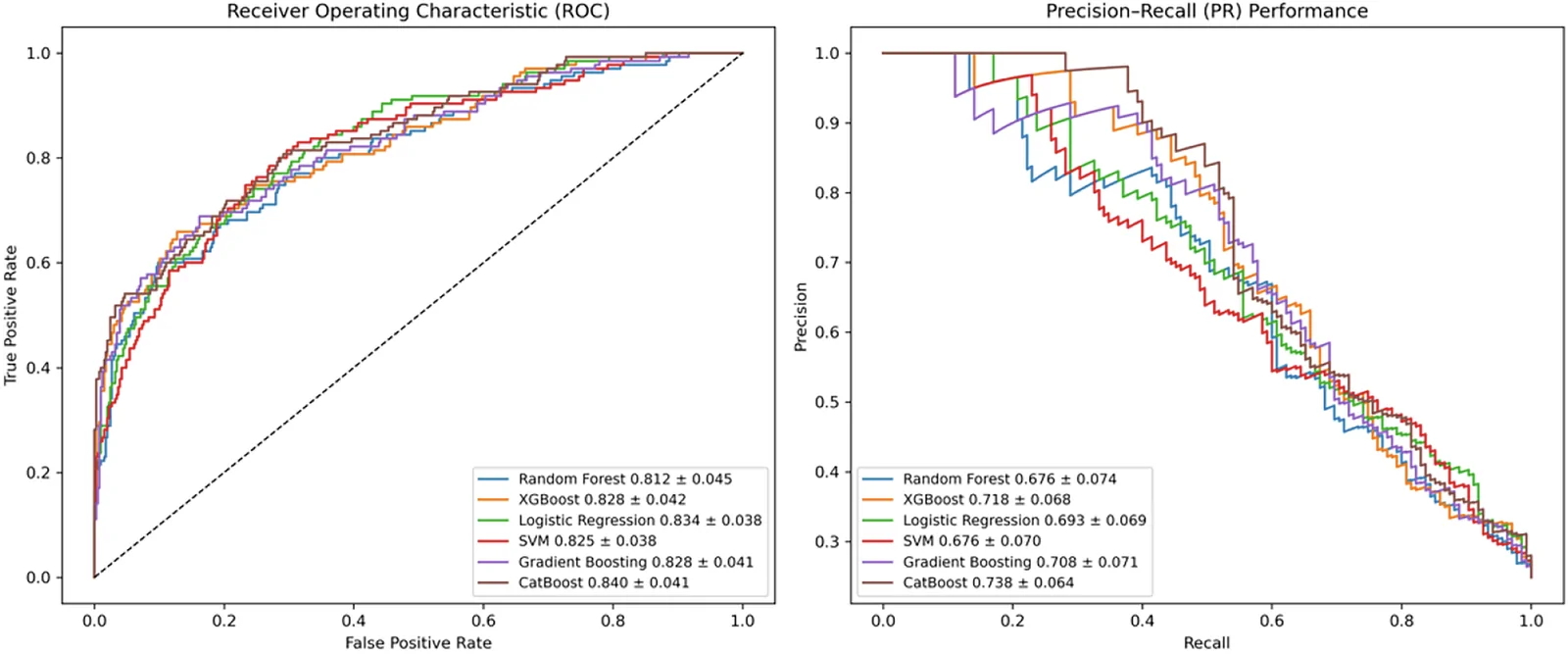
Performance curves for the six supervised machine learning models. Left: receiver operating characteristic (ROC) curves. Right: precision-recall (PR) curves. Legends report the area under the curve and 95% confidence interval. The dashed diagonal in the ROC panel denotes no-discrimination (AUROC = 0.5); The baseline AUPRC in the PR panel represents the event prevalence (≈0.22 positive rate). Across all models, CatBoost showed the strongest performance (AUROC = 0.840 ± 0.041; AUPRC = 0.738 ± 0.064). Other models ranged from AUROC 0.812 to 0.834 and AUPRC 0.676 to 0.718.

Following cohort characterization, six supervised machine learning models, CatBoost, XGBoost, Gradient Boosting, Logistic Regression, Random Forest, and Support Vector Machine (SVM), were evaluated on the same independent test set (Fig. 1). Using area under the precision-recall curve (AUPRC) as the primary performance metric, CatBoost achieved the highest value at 0.738 (95% CI: 0.670–0.798). This result was compared to the baseline PR AUC of 0.22, the lowest possible performance score for a model on a PR curve, equal to the proportion of positive samples in the dataset. As a secondary validation metric, the area under the ROC curve (AUROC) also ranked CatBoost highest at 0.840 (95% CI: 0.797–0.881). One-tailed permutation tests yielded *p* < 0.0001 for all models. Given the extremely low *p* values, even under conservative corrections, they would remain well below the 0.05 threshold, and therefore no formal correction was applied.

To examine threshold performance, we identified operating points optimized for the F1 and F2 thresholds for all models (Table 2). At the F1-optimal threshold (0.6468), CatBoost attained an F1 score of 0.6432, accuracy of 0.8508, precision of 0.7935, and recall of 0.5407. At the F2-optimal threshold (0.4000), the model achieved an F2 score of 0.7106, recall of 0.8148, precision of 0.4701, and accuracy of 0.7256, further confirming it as the strongest overall model. Given CatBoost’s consistent superiority across both AUPRC and AUROC, it was selected as the highest performing model for detailed interpretability and feature-level analyses.

**Table 2:** Test-set performance of each model at thresholds selected to maximize F1 (top) and F2 (*β* = 2, bottom)

Model statistics at F1-opt threshold								
Model	Threshold	Precision	Recall	F1 score	Accuracy	ROC AUC ± CI	PR AUC ± CI	*p* value
CatBoost	0.65	0.79	0.54	0.64	0.85	0.840 (0.801–0.877)	0.738 (0.674–0.797)	<0.0001
XGBoost	0.26	0.63	0.66	0.64	0.83	0.828 (0.784–0.868)	0.718 (0.647–0.781)	<0.0001
Gradient Boosting	0.33	0.73	0.57	0.64	0.84	0.828 (0.785–0.870)	0.708 (0.630–0.778)	<0.0001
Logistic Regression	0.66	0.69	0.56	0.61	0.83	0.834 (0.791–0.872)	0.693 (0.621–0.763)	<0.0001
Random Forest	0.57	0.67	0.60	0.63	0.83	0.812 (0.769–0.856)	0.676 (0.598–0.748)	<0.0001
SVM	0.25	0.54	0.70	0.61	0.78	0.825 (0.781–0.862)	0.676 (0.594–0.742)	<0.0001

Model statistics at F2-opt threshold								
Model	Threshold	Precision	Recall	F2 score	Accuracy	ROC AUC ± CI	PR AUC ± CI	*p* value
CatBoost	0.40	0.47	0.81	0.71	0.73	0.840 (0.801–0.877)	0.738 (0.674–0.797)	<0.0001
XGBoost	0.07	0.33	0.97	0.70	0.49	0.828 (0.784–0.868)	0.718 (0.647–0.781)	<0.0001
Gradient Boosting	0.11	0.37	0.88	0.69	0.60	0.828 (0.785–0.870)	0.708 (0.630–0.778)	<0.0001
Logistic Regression	0.33	0.40	0.90	0.72	0.64	0.834 (0.791–0.872)	0.693 (0.621–0.763)	<0.0001
Random Forest	0.40	0.43	0.80	0.68	0.69	0.812 (0.769–0.856)	0.676 (0.598–0.748)	<0.0001
SVM	0.18	0.47	0.83	0.72	0.72	0.825 (0.781–0.862)	0.676 (0.594–0.742)	<0.0001

Models are ranked by AUPRC, the primary evaluation metric. Reported values include the decision threshold, precision, recall, F1/F2 score, AUROC, and AUPRC, with 95% bootstrap confidence intervals and one-tailed permutation test *p* values.

**Fig. 2:**
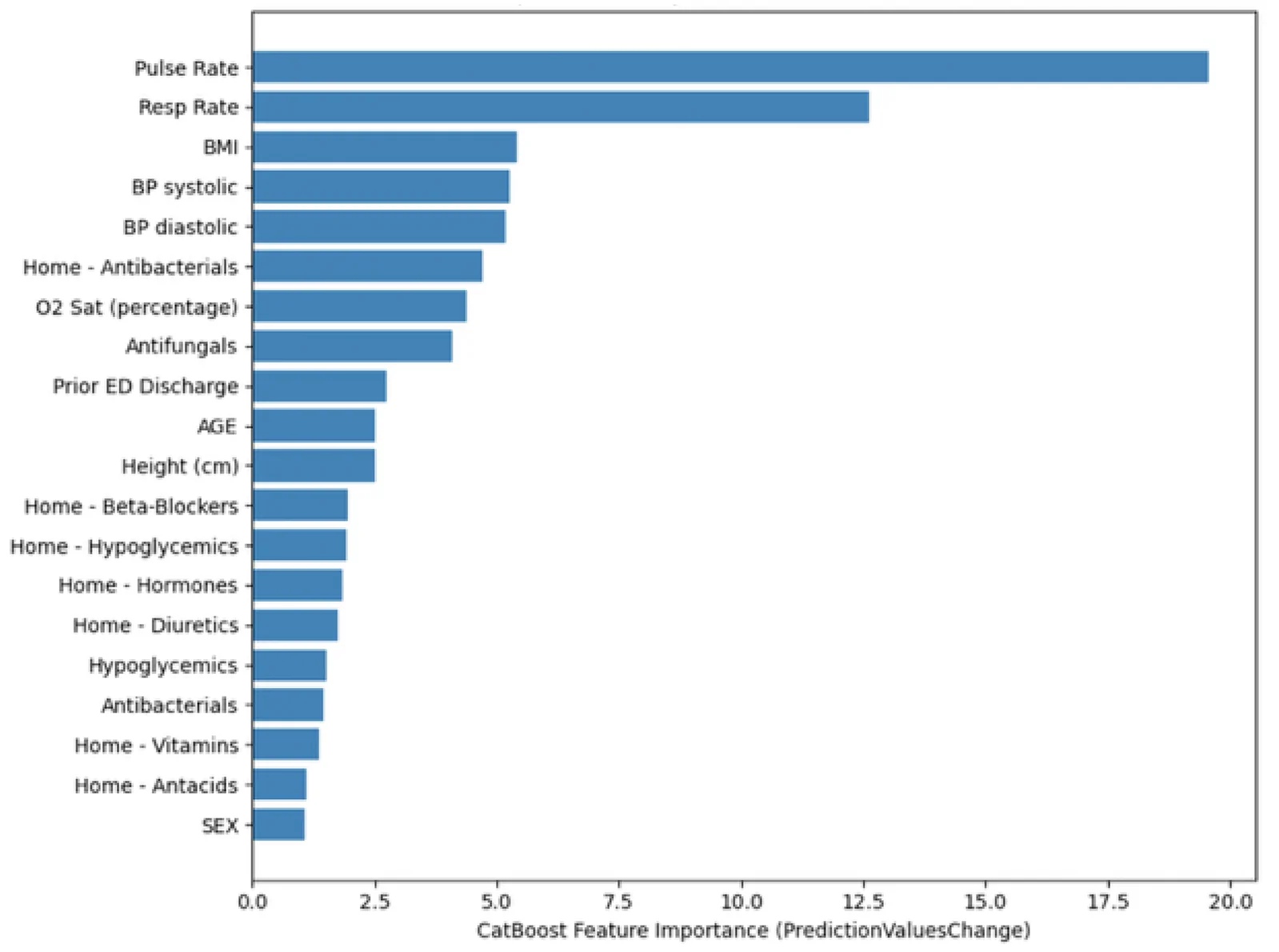
Horizontal bars display the top 20 predictors ranked by global importance in the CatBoost model. The *y*-axis lists features in order of importance, while the *x*-axis indicates feature importance based on the PredictionValuesChange metric (relative contribution to model prediction). Vital signs and BMI contributed most strongly to the model’s absolute risk estimate, followed by demographic variables and medication classes. Labels prefixed with “Home -” denote medications documented as home medications prior to admission; labels without the prefix refer to medications prescribed on the day of discharge

We used CatBoost’s PredictionValuesChange metric to assess global feature importance in the model. Importance values were calculated using the held-out test set and normalized to a 100% scale across all features. By this measure, Pulse Rate (19.55), Respiratory Rate (12.6), body mass index (BMI), systolic blood pressure (5.40), diastolic blood pressure (5.26), Home-Antibacterials (4.69), O₂ Saturation (4.38), and Antifungals (4.10) were identified as the highest-ranked predictors (Fig. 2; Supplementary Table S1).

**Fig. 3:**
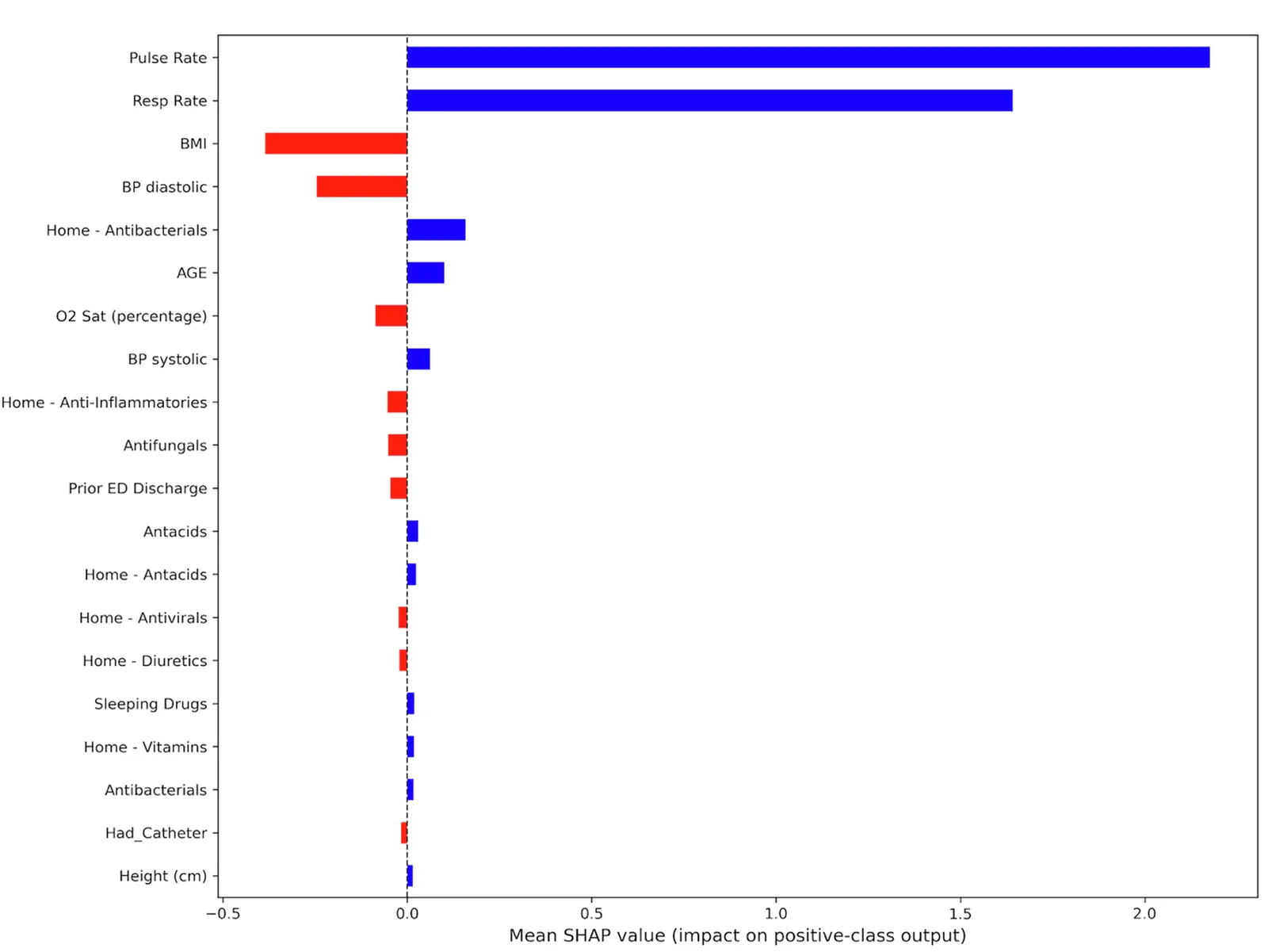
Directional mean SHAP values for the top 20 features in the CatBoost model. Horizontal bars represent each feature’s average contribution to the predicted risk of deterioration across the test set. The *x*-axis shows mean SHAP values, ordered by magnitude on the *y*-axis. Blue bars denote features that increase predicted risk, red bars denote features that reduce it, and the dashed vertical line marks zero contribution. Pulse rate and respiratory rate show the strongest positive influence, whereas diastolic blood pressure and BMI provide the strongest negative signals.

We used mean SHapley Additive exPlanations (SHAP) values for the positive class to assess directional feature influence in the model (Fig. 3; Supplementary Table S2). Positive SHAP values indicate that a feature increases the predicted risk of deterioration, whereas negative SHAP values indicate that a feature reduces the predicted risk. The strongest positive contributions were from Pulse Rate (+2.18), Respiratory Rate (+1.64), Home-Antibacterials (+0.16), and Age (+0.10), while BMI (−0.39) and diastolic blood pressure (−0.25) showed the strongest negative contributions. Together with the PredictionValuesChange ranking, these results highlighted Pulse Rate, Respiratory Rate, BMI, and diastolic blood pressure as the most influential features, which were selected as the top features in the model used for further evaluation.

**Fig. 4:**
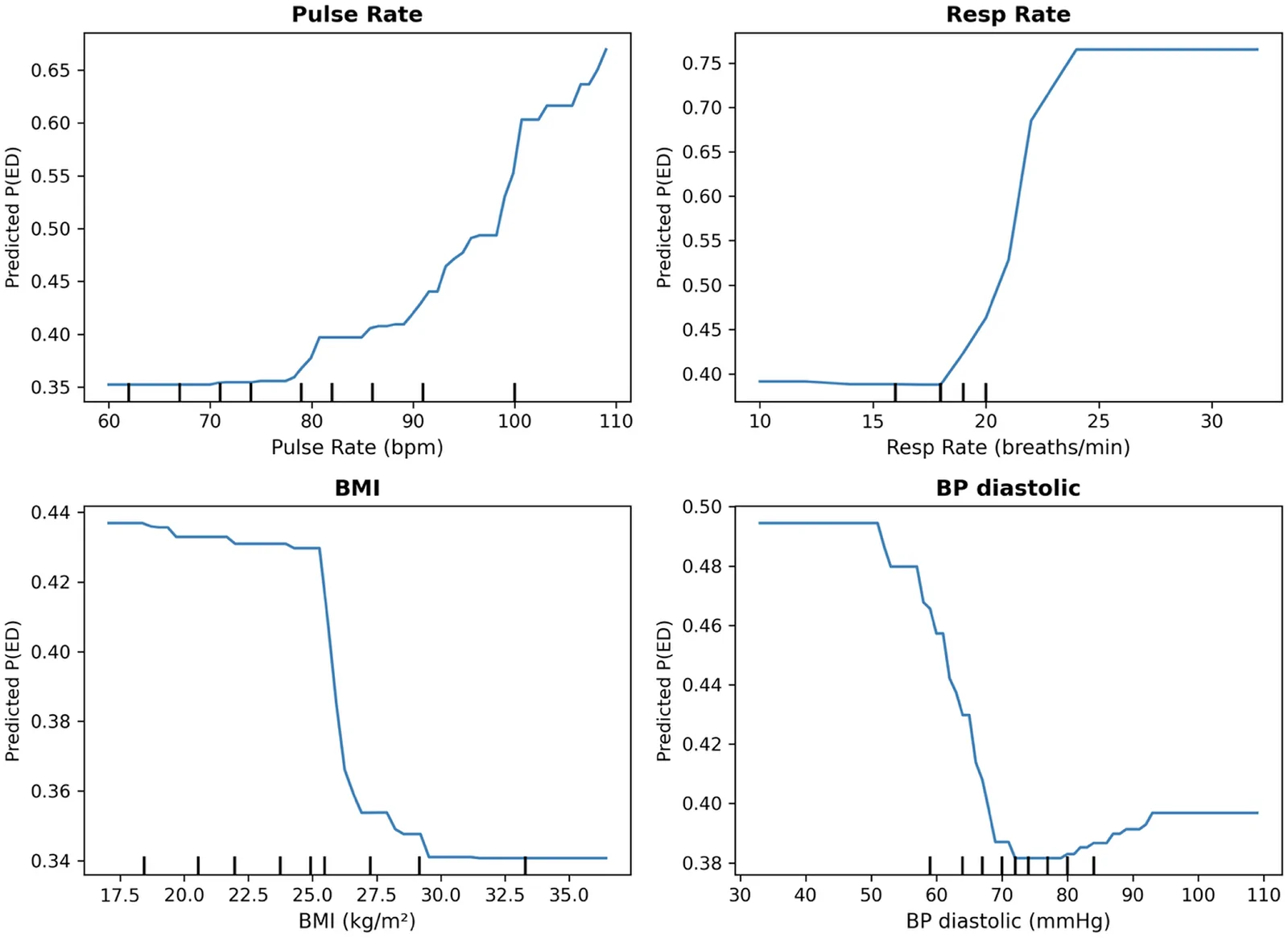
Partial-dependence plots for the four top features in the CatBoost model. Pulse Rate, Respiratory Rate, BMI, and diastolic blood pressure. Each panel shows the model-predicted probability of deterioration on the *y*-axis as the *x*-axis feature value varies, while the effects of all other predictors are averaged over the data. Short tick marks along the *x*-axis indicate where the evaluation data are most concentrated. Pulse Rate increases roughly monotonically; Respiratory Rate shows an S-shaped rise; BMI decreases and then plateaus; diastolic BP is highest risk at low values and declines to a stable low range as pressure increases.

Partial dependence plots (PDPs) for the top variables (Fig. 4) showed distinct patterns of association with predicted deterioration risk. For Pulse Rate, risk began to rise at approximately 80 bpm, with a steeper increase between 95 and 110 bpm. Respiratory Rate exhibited an S-shaped curve, with a sharp inflection between 20 and 25 breaths per minute, with plateaus at both low and high values. For BMI, predicted risk was high at low indexes and decreased beginning around 26 kg/m², stabilizing at lower levels above 30 kg/m². Diastolic blood pressure showed elevated risk at low values, declining steadily between roughly 55 and 70 mmHg, and remaining low thereafter with only a shallow rise above 80–90 mmHg. A more granular evaluation of the top variables can be found in Supplementary Fig. S1.

As a validation test to assess whether the model’s learned effects reflected clinically meaningful relationships rather than artifacts of multivariable interactions, we fit univariate logistic regression models for each of the top 20 SHAP-ranked features (Table 3; Supplementary Table S3). The four features with the largest directional SHAP impacts showed concordant directions in the univariate analysis: Pulse Rate (OR = 1.82, 95% CI: 1.66–1.99; *p* < 0.0001) and Respiratory Rate (OR = 1.87, 95% CI: 1.67–2.09; *p* < 0.0001) were associated with higher odds of deterioration, aligning with their positive mean SHAP contributions (+2.18 and +1.64, respectively). In contrast, BMI (OR = 0.82, 95% CI 0.72–0.93; *p* = 0.002) and diastolic blood pressure (OR = 0.72, 95% CI: 0.62–0.84; *p* < 0.0001) were associated with lower odds, matching their negative mean SHAP contributions (−0.39 and −0.25).

**Table 3:** Univariate odds ratios for the top 20 SHAP-ranked features, estimated from logistic regressions fit with one predictor at a time

Univariate odds ratios for the top 20 SHAP features			
Feature	Unit of change	OR (95% CI)	*p* value
Pulse Rate	Per 1 SD	1.82 (95% CI: 1.66–1.99)	*p* < 0.0001
Resp Rate	Per 1 SD	1.87 (95% CI: 1.67–2.09)	*p* < 0.0001
BMI	Per 1 SD	0.82 (95% CI: 0.74–0.91)	*p* < 0.0001
BP diastolic	Per 1 SD	0.72 (95% CI: 0.66–0.79)	*p* < 0.0001
Home—Antibacterials	1 vs 0	3.18 (95% CI: 2.61–3.86)	*p* < 0.0001
Age	Per 1 SD	1.02 (95% CI: 0.93–1.12)	*p* = 0.64
O2 Sat (percentage)	Per 1 SD	0.70 (95% CI: 0.64–0.76)	*p* < 0.0001
BP systolic	Per 1 SD	0.82 (95% CI: 0.75–0.90)	*p* < 0.0001
Home—Anti–Inflammatories	1 vs 0	1.14 (95% CI: 0.95–1.37)	*p* = 0.15
Antifungals	1 vs 0	3.09 (95% CI: 2.57–3.72)	*p* < 0.0001
Prior ED Discharge	1 vs 0	3.53 (95% CI: 2.81–4.44)	*p* < 0.0001
Antacids	1 vs 0	2.73 (95% CI: 2.09–3.61)	*p* < 0.0001
Home—Antacids	1 vs 0	2.39 (95% CI: 1.87–3.04)	*p* < 0.0001
Home—Antivirals	1 vs 0	2.28 (95% CI: 1.85–2.80)	*p* < 0.0001
Home—Diuretics	1 vs 0	2.66 (95% CI: 2.21–3.19)	*p* < 0.0001
Sleeping Drugs	1 vs 0	1.26 (95% CI: 1.05–1.51)	*p* = 0.01
Home—Vitamins	1 vs 0	4.69 (95% CI: 2.88–7.62)	*p* < 0.0001
Antibacterials	1 vs 0	3.03 (95% CI: 2.49–3.68)	*p* < 0.0001
Had Catheter	1 vs 0	2.34 (95% CI: 1.74–3.15)	*p* < 0.0001
Height (cm)	Per 1 SD	1.04 (95% CI: 0.95–1.15)	*p* = 0.37

Each row lists the feature, the unit of change used in the regression (e.g., per 1 SD for continuous variables or 1 vs. 0 for binary indicators), and its odds ratio (OR) for deterioration with 95% confidence intervals (CI) and *p* values. ORs greater than 1 indicate higher odds of deterioration, while ORs less than 1 indicate lower odds.

To quantify the concentration and redundancy of predictive signal, we performed ablation experiments (Table 4; Supplementary Figs. S2 and S3; Supplementary Tables S4 and S5). A model trained using only the top four features achieved an AUPRC of 0.582 (95% CI: 0.502–0.652) and an AUROC of 0.742 (0.691–0.790), while a model trained on all remaining features excluding these four achieved an AUPRC of 0.655 (0.582–0.728) and an AUROC of 0.795 (0.753–0.840). For reference, the full CatBoost model achieved an AUPRC of 0.738 (0.670–0.798) and an AUROC of 0.840 (0.797–0.881). The Top-4 Only features model performed significantly worse, as its confidence intervals did not overlap the full model (AUPRC upper bound 0.652 < 0.670; AUROC upper bound 0.790 < 0.797). In contrast, the All Except Top-4 features model showed overlapping intervals with the full model (AUPRC overlap 0.670–0.728; AUROC overlap 0.797–0.840), indicating that the predictive signal was distributed across the broader feature set. Both ablation configurations underperformed the full CatBoost model overall.

**Table 4:** Performance of two ablation configurations on the test set: top-4 only (pulse rate, respiratory rate, BMI, diastolic BP) and all except top-4 features (all remaining predictors)

CatBoost ablation summary						
Feature set	ROC AUC (95% CI)	PR AUC (95% CI)	F1 score	F2 score	*p* value	ΔPR AUC vs full
All except the top 4	0.795 (0.752–0.840)	0.655 (0.578–0.737)	0.59	0.67	*p* < 0.0001	−0.083
Top 4 features only	0.742 (0.687–0.788)	0.582 (0.502–0.658)	0.52	0.66	*p* < 0.0001	−0.156
All features (full)	0.840 (0.801–0.877)	0.738 (0.674–0.797)	0.64	0.71	*p* < 0.0001	+0.000

For each configuration, results are reported at thresholds maximizing F1 and F2, including threshold, precision, recall, accuracy, PR AUC with 95% confidence intervals, ROC AUC with 95% confidence intervals, and one-tailed permutation *p* values.

Finally, to better understand the contribution of the 394 repeat patients, that is, patients who were admitted and discharged multiple times within the study period, representing approximately 923 out of 2711 discharges, or approximately 35% of our data, we ran our CatBoost model without repeat patients. There was a notable decrease in performance, with AUPRC decreasing to 0.396 (from 0.738) and AUROC decreasing to 0.769 (from 0.840). For comparison, we ran our model with the same number of discharges removed at random, and found a much more modest decrease in performance, with AUPRC of 0.705 and AUROC of 0.833. Overall, this does suggest that the model relies disproportionately on patients with repeat discharges, possibly because there is more homogeneity in their repeat clinical presentations than in the total set of discharges.

## Discussion

This study represents a novel application of AI for the prediction of patient deterioration in the post-acute setting. While there is growing literature on similar AI applications in the acute-care context, it has not been well explored in post-acute hospitals.

A primary finding is that robust performance was achieved despite multiple clinical and technical barriers that might have limited model development. In the post-acute context, there is less rich physiologic data available than in acute-care. We were restricted to choosing a discrete variable (discharge to the ED) to represent deterioration, which was both clinically meaningful in our context and could also be pulled reliably from our legacy EMR system. Features that might be associated with risk of deterioration, such as vital signs, are monitored less frequently in post-acute care. Elements of care specific to post-acute hospitals, such as data related to rehabilitative care, which theoretically might have been associated with risk of deterioration, were documented on paper or were not feasible to pull from our EMR and, therefore, could not be used. Despite this, as evidenced by the high AUPRC and other related measures for CatBoost and the other tested models, the available data clearly predicted the risk of deterioration well. This provides a clear, novel proof of concept that available, discrete-data elements are sufficient for meaningful prediction of risk of clinical deterioration in the post-acute context.

Second, the physiologic plausibility of the pattern of contribution of the model’s features to risk of deterioration provides evidence that our findings are likely sound, and potentially represents novel evidence that the key predictors of deterioration in acute care hold in post-acute environments. Importantly, these findings are statistical associations only and cannot distinguish clinician behavior in response to clinical concerns like abnormal vital signs from true intrinsic deterioration. Still, whether capturing clinician behavior or true deterioration or both, alignment with known predictors in other environments at minimum provides additional evidence that these results are not mere statistical aberration, and potentially do suggest the utility of familiar predictive features in post-acute contexts, though this does require additional study to explore further. CatBoost feature importance and SHAP analysis identified vital signs as key contributors to risk of deterioration with directionally coherent associations: higher pulse and respiratory rates were associated with increased predicted risk, while higher diastolic blood pressure and BMI were associated with decreased predicted risk. This is consistent with established clinical understanding^1^. Heart rate, respiratory rate, and blood pressure are essential clinical measures of basic physiologic function; in acute-care settings, most patients who experience severe deterioration have abnormal vital signs in the hours beforehand^2,3,8^. PDPs for the top features (Fig. 4) showed further alignment between our findings and known predictors of risk in acute care. In our analysis, higher pulse and respiratory rates were associated with increased predicted risk, while higher diastolic blood pressure was associated with decreased risk, exactly as expected in common causes of deterioration in acute-care, such as systemic inflammation, sepsis, shock, and other acute, life-threatening conditions^9–11^.

BMI showed a more complex relationship with deterioration risk. Very low BMI is frequently associated with advanced illness such as cancer, significant frailty, or psychosocial vulnerability, all of which increase the likelihood of clinical decline^12,13^; it is also possible that very low BMI increases clinicians’ likelihood of escalation of care, given the patient’s appearance of being medically unwell. Although extremely high BMI may also pose risk through other mechanisms, the small number of patients in this category makes it difficult for the model to predict high risk for these individuals. The overall shape of the BMI risk curve in our data is broadly consistent with expectations, with higher risk at low BMI values; however, we would expect the curve to rise again at higher BMI values if more patients in this range were available. The observed curve was also shifted toward the middle range, likely due in part to data quality limitations. While vital signs were measured directly by nursing staff and ED discharges were objectively recorded, heights and weights were often patient-reported or measured using non-standardized equipment such as non-zeroed bed scales. In addition, there were a small number of missing height values, which we imputed using the median; this approach reduced variability and may have further concentrated BMI values toward the middle range, limiting the curve’s representation at both extremes. Despite these limitations, the general pattern of association remains consistent with expectations and is likely meaningful.

Our results align closely with established deterioration scoring systems. The National Early Warning Score identifies pulse rate above 90 bpm and respiratory rate above 20 breaths/min as thresholds for increased risk, both of which correspond closely to the inflection points in our PDPs (~80–95 bpm with a steeper increase to ~110 bpm for pulse rate; ~20–25 breaths/min for respiratory rate). The Systemic Inflammatory Response Syndrome criteria include the same pulse and respiratory rate thresholds^14^. The quick Sequential Organ Failure Assessment score identifies respiratory rate at or above 22 breaths/min as a risk criterion and includes a systolic blood pressure threshold^15,16^. While these scoring systems were developed and validated in acute-care populations, our findings suggest that similar physiologic thresholds may be associated with risk of deterioration in the post-acute setting. This raises the possibility that these or similar scoring systems could be validated in the post-acute context.

Univariate comparisons reinforced that our findings represent real clinical phenomenology, rather than statistical model features. When considered alone, the top predictors—pulse rate, respiratory rate, diastolic blood pressure, and BMI—showed the same directional associations as their mean SHAP contributions, reinforcing the plausibility of their clinical interpretation. Expectedly, for other features with smaller SHAP values, the features at times diverged. For instance, in the univariate analysis, “Prior ED Discharge” was strongly associated with increased deterioration risk (OR = 3.53, 95% CI: 2.81–4.44; *p* < 0.0001), which is clinically intuitive as previous deterioration is expected to increase risk of future deterioration, whereas its mean SHAP contribution appeared slightly protective (−0.046). This and similar mismatches are expected, given that univariate odds ratios capture raw marginal associations, while SHAP values describe each feature’s conditional contribution in the context of multiple correlated variables. Taken together, however, alignment of SHAP contributions, univariate correlations, and PDP plots with physiologic expectations helps distinguish what is a convincingly genuine signal from the contribution of these variables from artifacts of modeling or confounding.

Finally, building on this, our ablation experiments tested how much predictive signal resides in the most prominent features versus the complete set. The ablation analyses demonstrated that the top features, namely pulse rate, respiratory rate, diastolic blood pressure, and BMI, can themselves be meaningfully predictive. However, they also showed that the broader feature set meaningfully strengthens prediction. When all features were included, the CatBoost model performed best; when the top four predictors were excluded, the model’s performance remained close to the full CatBoost model, with overlapping confidence intervals, indicating that additional information was captured by medications, prior encounters, presence of a bladder catheter, and demographics. This distributed signal increases the likelihood of detecting patients at risk who may not present with classic vital sign abnormalities. In post-acute care, where physiologic monitoring is less frequent and deterioration is more difficult to recognize, this broader modeling approach provides an important advance. By integrating both well-established predictors and secondary signals, the machine learning model increases its performance and demonstrates a pathway for leveraging EMR data in settings where predictive tools are still underdeveloped.

Taken together, these results show that this novel application of AI in a post-acute hospital potentially predicts the risk of deterioration in a way that is both meaningful and physiologic, not before shown in other studies in this care setting.

There are a number of limitations of this study. This work originated in a quality improvement project at our hospital site to improve escalation of care. As such, this is a single-center study, and further studies at similar sites would further validate our findings. Further, the total number of patient discharges was sufficient for the models we used, but limited the total number of features we could include. Given their limited clinical use in post-acute settings, it is also difficult to evaluate whether a simpler multivariate model, analogous to early warning systems used in acute care, would perform similarly to this CatBoost model, but without the need for burdensome AI complexity.

There are important limitations to this work as a result of its being a retrospective statistical study. As noted above, clinician behavior where care is escalated in response to the variables captured in this study cannot be distinguished from intrinsic deterioration associated with those variables: for instance, a clinician may be more likely to send a patient to the ED if they have abnormal vital signs, rather than the vital signs portending deterioration per se. An important potential limit of this study is the “early warning paradox”^17^, namely, where a retrospective predictive model misses cases where deterioration was avoided because of successful clinical intervention. This is potentially problematic in our study, given our reliance on a workflow, rather than a physiologic variable, for defining deterioration. Despite this, the early warning effect is expected to be much more minor in the post-acute setting, such as our hospital. There is little opportunity to stabilize an unwell patient in post-acute care, given the limited availability of interventions to manage clinical deterioration and the limited staff to monitor safety. As such, the threshold for escalation is much lower, and the primary mechanism for managing expected deterioration, even when caught early, is transfer to the ED.

There is potential information leakage in our study, complicated by limitations of our data quality. Nurses may check vitals more frequently for a patient whom they are concerned may need ED transfer, and therefore, the most recent vital signs prior to discharge may reflect a component of clinical concern independent of what was gleaned from vitals themselves. It is difficult to control for this in the current model. Both vital sign and time of discharge data are manually entered into our EMR asynchronously at variable intervals depending on hospital staff workflow needs; as such, it is not possible to determine the timing between the last recorded vital signs and the timing of discharge to the ED. Future studies will look at vital signs changes over multiple measurements and prior to the day of discharge to try to account for this effect. Furthermore, as noted above, there are limits to the data quality of our BMI measurements.

Finally, further work is needed to test the implementation of this work into real workflows to improve patient safety, which is the next direction our group is planning.

## Methods

### Setting, approach, and defining the target variable

This study was conducted at Hennick Bridgepoint Hospital (HBH), a 474-bed urban, adult, post-acute hospital serving a broad population across all major streams of rehabilitation and complex continuing care. The hospital uses a 19-year-old legacy EMR system; only discrete data can be pulled. A collaboration of human-factors specialists, clinical application specialists, and physicians working at HBH worked together to identify discrete data elements appropriate for analysis. These were identified through: analysis of clinical and EMR workflows to reveal which discrete data elements were present in the EMR; validation of which of these elements were felt to be reliable measures of real-world parameters; verification of which elements were feasible for data pull; and fine-tuning and data cleaning to ensure the elements were reliable and clinically meaningful. These elements will be described in more detail below.

Discharge to the emergency department (ED) was selected as the measure for patient deterioration. Clinically, patients at HBH who deteriorate and are felt to require an escalation of care beyond the acuity manageable at HBH are sent to an acute-care hospital ED for evaluation. Any patient whom the ED team does not feel warrants acute-care admission is sent back to HBH to continue their care; this reflects the majority of patients sent to the ED from HBH. Only patients whom the ED team feels require admission to acute care are admitted to their facility, and are therefore deemed discharged to the ED from HBH. Importantly, this means that discharge to the ED reflects not only the clinical behavior of the HBH clinical team, who chose to send the patient to the ED, but also the receiving ED team, whose separate assessment determined that the patient’s care needs did indeed warrant admission. As such, while discharge to the ED does reflect a workflow, rather than physiologic, variable, it is not specific to the clinical behavior of a single team, and does select for the patients most likely to have a true increase in care needs. Therefore, discharge to the ED was defined as the positive class for our binary target variable, with other discharge destinations defined as the negative class.

With the data elements determined, we conducted a retrospective analysis of 2,876 patient discharges from HBH between April 2024 and July 2025. Internal transfers and in-hospital deaths were excluded. With respect to in-hospital deaths, there were 64 in the total group of patient discharges. The vast majority of these were known to be expected deaths in the context of palliative care. There were 45 deaths (i.e., 70% of total deaths) on the palliative care unit during this time period, as well as many other patients who received terminal palliative care on other units, though the specific number is not available. As our dataset could not distinguish palliative from unexpected deaths, but the former group was by far the largest, and the total remaining number of deaths was small relative to our total number of discharges, we excluded deaths from our analysis. This left 2711 discharges for analysis. Discharges to the ED accounted for 22% of the cohort. A total of 394 patients were repeat cases, meaning they had multiple discharge events during the study period. Each discharge was treated as a separate event and was statistically studied only in relation to patient variables from the corresponding admission, rather than other admissions for the same patient.

### Features used for the predictive model

Five feature classes were extracted from the EMR^18,19^. One, demographics, including age, sex, height, and BMI; weight was excluded given its linear relationship to BMI, with BMI felt to be more clinically meaningful. Two, vital signs, including pulse rate, respiratory rate, systolic blood pressure, diastolic blood pressure, and oxygen saturation, all using the most recent available measurement prior to discharge. Three, previous discharge to the ED, meaning that the patient under consideration for the current discharge had been discharged to the ED while a patient at HBH on an earlier admission. Fourth, presence of a urinary bladder catheter at any time over the admission, given that more granular data reflecting the presence of a urinary bladder catheter at the time of discharge specifically was not felt to be reliably available in the EMR. Fifth, prescribed medications, including a category for each of the medications prescribed at home and medications prescribed on discharge. For this final category, each specific medication was assigned to one of 41 FDA-defined general drug categories to manage the number of inputs into the model^20^. We did also pull laboratory results, but these were ultimately excluded given multiple data issues, including: absent laboratory data for a large subset of patients (only 84% of patients had even the most common laboratory tests); large variability between patients in the frequency of tests, ranging from multiple times per week to once every several months to never; large variability between patients with respect to the timing of the most recent laboratory tests and the day of discharge, ranging from same day to months prior; risk of data leakage given laboratory tests were more likely to be ordered when a clinician suspected deterioration. All continuous variables in the above features were retained in their respective units, whereas other features were binary coded. All features were mapped to each other using a unique patient identifier present throughout the pulled datasets.

### Model selection

We selected six supervised machine learning algorithms to balance interpretability and predictive power: logistic regression (LR), Support Vector Machine (SVM), random forest (RF), gradient boosting, XGBoost, and CatBoost. These models were chosen because they span a range of linear and non-linear approaches and have been widely applied in healthcare predictive modeling, including in acute care settings^21–23^. This selection allowed us to test performance across diverse algorithmic families when applied to structured EMR data^24,25^.

### Model development and tuning

The above set of predictor features and the binary outcome variable of discharge to the ED were compiled for all included patient records. Data were partitioned into training (80%) and test (20%) sets using a consistent random state to ensure reproducibility.

The only feature for which data values were missing was BMI, for which only approximately 9% of values were missing. Given the low percentage of missing values, we did not feel that this was likely to significantly impact model performance. For linear models such as LR and SVM, missing values were imputed using a mean-based SimpleImputer, and continuous variables were standardized with a StandardScaler^26^ to ensure proper model performance. Tree-based models, including random forest, gradient boosting, XGBoost, and CatBoost, did not require feature scaling. XGBoost and CatBoost can handle missing values natively, while random forest and gradient boosting require the same mean-based imputation used for the linear models. Applying a consistent imputation method across all models ensured that any observed differences in performance reflected the models themselves rather than differences in missing data handling.

Hyperparameters for each model were tuned using *GridSearchCV*^27^ with stratified 5-fold cross-validation^28^ to maintain the 22% positive class proportion within each fold. The F1 score was used as the primary optimization metric to balance precision and recall in this imbalanced dataset^29^. The optimal hyperparameters identified during tuning were then used to retrain each model on the full training set prior to final evaluation.

### Threshold optimization

Probability estimates from each model on the held-out test set were used to construct precision-recall curves. From these curves, we identified two operating thresholds for each model: one that maximized the F1 score and one that maximized the F2 score. The F1 score is the harmonic mean of precision and recall with equal weighting, reflecting scenarios where false positives and false negatives carry similar clinical importance. The F2 score, in contrast, places four times more weight on recall than precision, prioritizing the identification of as many true deterioration cases as possible at the expense of generating more false alarms. This weighting aligns with the clinical imperative in post-acute care to avoid missed detections of patient deterioration, where the cost of a false negative is substantially higher than that of a false positive^30,31^.

Models were evaluated at both thresholds to understand trade-offs relevant to potential clinical applications.

### Evaluation

Model performance was assessed using confusion matrices, precision, recall, F1, and F2 scores, as well as precision-recall area under the curve (PR AUC) and receiver operating characteristic area under the curve (ROC AUC). PR AUC was the primary performance metric given the imbalanced outcome (22% positive class) and the clinical priority of identifying patients who require transfer to the ED^32^. It captures the trade-off between recall and precision in the positive (deterioration) class, providing a direct measure of the model’s ability to detect true positive deterioration events while limiting false positives. ROC AUC was included as a secondary metric to facilitate comparison with prior studies, where it remains a common benchmark, despite its tendency to be inflated in imbalanced datasets by easier classification of the majority negative class. For each metric, 95% confidence intervals were calculated using 1000 stratified bootstrap resamples of the test set, and statistical significance between models was evaluated using a one-tailed permutation test with 1000 permutations.

### Model interpretability

We further analyzed the CatBoost model, which was determined to be the best-performing model based on evaluation metrics (see above). Feature importance was first assessed using CatBoost’s *PredictionValuesChange* metric, which summarizes the average absolute change in predicted risk when a split uses a given feature, aggregated across all trees. Importances were calculated on the held-out test set and normalized to a 100% scale across all features, identifying those contributing most in magnitude to the model’s predictions.

We paired feature importance with SHAP to capture both the magnitude and direction of each predictor’s influence. Directional mean impact SHAP values were calculated to quantify each feature’s total marginal contribution to the model’s predictions, incorporating all interactions with other variables. Across the cohort, a positive SHAP value indicated that the feature increased the predicted probability of deterioration relative to the model’s baseline, while a negative value indicated a corresponding decrease.

To examine true modeled trends and validate the SHAP findings, we generated PDPs for the top-ranked features. PDPs depict the model’s average predicted probability of deterioration as a single feature varies, while holding all other variables constant. PDPs allowed us to understand the trends for the top features to verify that the model aligned with clinical expectations and validation.

We also created SHAP ribbon plots, which show the average SHAP value in bins of the feature’s actual observed values. Since SHAP values are computed per patient while accounting for all other features, the ribbon preserves the context-dependent effect of that feature. Pairing SHAP ribbon plots with PDPs allowed us to confirm that feature effects observed in the exact patient-specific contexts (SHAP) are consistent with the overall marginal trends in predicted probability (PDP), supporting both the plausibility and validity of the model’s behavior.

To complement model-based explanations, we estimated each predictor’s stand-alone association with the outcome using univariable odds ratios (ORs). Since the outcome was coded as *y* = 1 for a discharge to the ED, OR > 1 indicates higher odds of discharge, and OR < 1 indicates lower odds of discharge. For continuous predictors, we fit univariable logistic regressions after standardizing the predictor (per 1 SD), so effect sizes are comparable across scales. For binary predictors, we computed 2 × 2 odds ratios. Analyses used the combined train/test set; for each feature, rows missing that feature were excluded only from its analysis. These univariable ORs are reported descriptively and interpreted alongside SHAP/PDP results to place single-feature associations within multivariable patterns and clinical context.

Finally, we performed ablation experiments by (i) retraining the model using only the top SHAP-ranked features and (ii) excluding these features entirely. These tests evaluated whether predictive performance could be maintained with a reduced feature set and whether other discrete EMR variables provided incremental value beyond the top-ranked predictors.

### Ethics statement

This study was approved by the Sinai Health Research Ethics Board, REB # 1472.

## Supplementary information

## Data Availability

The datasets generated and analyzed during the current study are not publicly available for patient privacy reasons, but are available from the corresponding author on reasonable request.

## Code availability

The underlying code for this study may be made available to qualified researchers on reasonable request from the corresponding author.

## References

[1] Hillman, K. M. et al. Antecedents to hospital deaths. *Intern. Med. J.* **29**, 343–348 (1999). 10.1046/j.1445-5994.2001.00077.x 11529588

[2] Subbe, C. P. et al. Machine learning models for predicting deterioration in hospitalized patients: a systematic review. *J. Clin. Monit. Comput.* **35**, 1153–1165 (2021).

[3] Churpek, M. M. et al. Multicenter comparison of machine learning methods and conventional regression for predicting clinical deterioration on the wards. *Crit. Care Med.* **41**, 1719–1728 (2013). 26771782 10.1097/CCM.0000000000001571 PMC4736499

[4] Churpek, M. M., Yuen, T. C., Park, S. Y., Meltzer, D. O. & Edelson, D. P. Using electronic health record data to predict clinical deterioration in hospitalized patients. *Crit. Care Med.* **44**, 1632–1641 (2016).27428135

[5] Turner, J. R. et al. Artificial intelligence and pathology: diagnostic opportunities and regulatory challenges. *Mod. Pathol.* **37**, 1–14 (2024).

[6] Alvarado, C., Zook, K. & Henry, J. Electronic Health Record Adoption and Interoperability among U.S. Skilled Nursing Facilities in 2016. In: ASTP Health IT Data Brief [Internet]. Washington (DC): Office of the Assistant Secretary for Technology Policy; 2012. Vol. 39 Available from: https://www.ncbi.nlm.nih.gov/books/NBK610657/ (2017). 39808053

[7] Hakkarainen, T. W. et al. Outcomes of patients discharged to skilled nursing facilities after acute care hospitalizations. *Ann. Surg.* **263**, 280–285 (2016). 26445466 10.1097/SLA.0000000000001367 PMC4706779

[8] Kause, J. et al. A comparison of antecedents to cardiac arrests, deaths, and emergency intensive care admissions in Australia and New Zealand, and the United Kingdom—the ACADEMIA study. *Resuscitation* **62**, 275–282 (2004). 15325446 10.1016/j.resuscitation.2004.05.016

[9] Sproston, N. R. & Ashworth, J. J. Role of C-reactive protein at sites of inflammation and infection. *Front. Immunol.* **9**, 754 (2018). 29706967 10.3389/fimmu.2018.00754 PMC5908901

[10] Chen, L. M. et al. Failure to rescue after surgery in the United States: a population-based study. *Ann. Surg.* **262**, 660–666 (2015).26366546

[11] Escobar, G. J. et al. Early detection of impending physiologic deterioration using electronic medical record data. *BMJ Qual. Saf.* **26**, 512–520 (2017).

[12] Allison, D. B. et al. Body mass index and all-cause mortality: a review. *JAMA Intern. Med.* **157**, 749–757 (1997).

[13] Fearon, K. C. H. et al. Definition and classification of cancer cachexia: an international consensus. *Lancet Oncol.* **12**, 489–495 (2011). 21296615 10.1016/S1470-2045(10)70218-7

[14] Bone, R. C. et al. American College of Chest Physicians/Society of Critical Care Medicine Consensus Conference: definitions for sepsis and organ failure and guidelines for the use of innovative therapies in sepsis. *Crit. Care Med*. **20**, 864–874 (1992). 1597042

[15] Singer, M. et al. The Third International Consensus Definitions for Sepsis and Septic Shock (Sepsis-3). *JAMA* **315**, 801–810 (2016). 26903338 10.1001/jama.2016.0287 PMC4968574

[16] Kipnis, P. et al. Development and validation of an electronic medical record-based alert score for detecting patient deterioration. *BMJ Qual. Saf.* **25**, 870–879 (2016). 10.1016/j.jbi.2016.09.013 PMC5510648 27658885

[17] Logan Ellis, H. et al. The early warning paradox. *npj Digit. Med.* **8**, 81 (2025). 39900787 10.1038/s41746-024-01408-x PMC11790821

[18] Mohammed, S. et al. A deterioration prediction model was developed using patient demographics, ward-based observations, laboratory values, and their trends. *J. Am. Med. Inform. Assoc.* **28**, 1593–1603 (2021).

[19] Escobar, G. J. et al. Early detection of impending physiologic deterioration among patients who are not in intensive care. *BMJ Qual. Saf.* **22**, 512–520 (2013). 10.1002/jhm.1929 22447632

[20] U.S. Food and Drug Administration. General drug categories. https://www.fda.gov/drugs/investigational-new-drug-ind-application/general-drug-categories (accessed 2025).

[21] Shickel, B. et al. Deep learning for patient-specific prediction of clinical events from electronic health records. *Artif. Intell. Med.* **147**, 102818 (2024).

[22] Islam, M. M. et al. Machine learning models for predicting hospital readmission: a systematic review. *PLoS ONE* **16**, e0247943 (2021).33684164

[23] Abdar, M. et al. E-CatBoost: An efficient machine learning framework for predicting ICU mortality using the eICU collaborative research database. *Comput. Methods Programs Biomed.* **225**, 107023 (2022). 10.1371/journal.pone.0262895 PMC9070907 35511882

[24] Escobar, G. J. et al. Early detection of impending physiologic deterioration among patients who are not in intensive care: development of predictive models using data from an automated electronic medical record. *J. Hosp. Med.* **7**, 345–352 (2012). 22447632 10.1002/jhm.1929

[25] Yu, Y. et al. Predicting acute clinical deterioration with interpretable machine learning to support emergency care decision making. *Sci. Rep.* **13**, 17124 (2023). 37604974 10.1038/s41598-023-40661-0 PMC10442440

[26] Lee, S. et al. Machine learning and prediction models in post-acute care: systematic review. *Med. Devices Sens.* **6**, e10160 (2023).

[27] McCoy, A. B. et al. Machine learning models for early warning of clinical deterioration: a systematic review. *J. Am. Med. Inform. Assoc.* **28**, 1821–1832 (2021).

[28] Wang, X. et al. Data preprocessing techniques for machine learning in healthcare: a systematic review. *Front. Digit. Health* **2**, 136 (2024).

[29] Zhao, H. et al. Developing and validating a prediction model for early detection of patient deterioration in the ICU. *Crit. Care Explor.* **5**, e0931 (2023).37303944

[30] Liu, Y. et al. Comparative study of ML algorithms for predicting adverse outcomes in emergency settings. *Sci. Rep.* **13**, 9217 (2023). 37280304 10.1038/s41598-023-34556-3 PMC10244345

[31] Singh, K. et al. Evaluating F-scores for clinical prediction models: comparative performance of F1 and F2 metrics. *BMJ Health Care Inform.* **31**, e101088 (2024).

[32] Chen, W. et al. Predicting patient deterioration with multimodal EHR data using interpretable deep learning models. *npj Digit. Med.* **8**, 112 (2025).39966675

