# Supplementary material for: A novel use of AI for prediction of clinical deterioration in a post-acute hospital
Source: Npj Health Syst. 2026 Sep 2;3:84. doi: 10.1038/s44401-026-00145-5 (PMC13538516; doi:10.1038/s44401-026-00145-5)
Supplement: Supplementary file 1 [file 44401_2026_145_MOESM1_ESM.pdf]

Supplementary Materials

Feature	Importance
Pulse Rate	19.55
Resp Rate	12.6
BMI	5.4
BP systolic	5.26
BP diastolic	5.17
Home – Antibacterials	4.69
O2 Sat (percentage)	4.38
Antifungals	4.09
Prior ED Discharge	2.74
AGE	2.5
Height (cm)	2.49
Home - Beta-Blockers	1.94
Home – Hypoglycemics	1.91
Home – Hormones	1.85
Home – Diuretics	1.74
Hypoglycemics	1.51
Antibacterials	1.46
Home – Vitamins	1.37
Home – Antacids	1.09
SEX	1.06
Antacids	1.03
Home – Anticonvulsants	1.00
Home – Antivirals	0.95
Beta-Blockers	0.87
Home - Anti-Inflammatories	0.82
Home – Sedatives	0.77
Sleeping Drugs	0.68
Home – Antihistamines	0.68
Diuretics	0.65

Antipsychotics	0.64
Immunosuppressives	0.55
Home – Antipsychotics	0.53
Corticosteroids	0.52
Antihypertensives	0.46
Anti-Inflammatories	0.46
Home – Antiarrhythmics	0.45
Bronchodilators	0.45
Sedatives	0.43
Hormones	0.42
Had_Catheter	0.42
Home – Antihypertensives	0.32
Home - Sleeping Drugs	0.32
Antianxiety Drugs	0.31
Antiemetics	0.28
Antipyretics	0.27
Home - Antianxiety Drugs	0.27
Home – Laxatives	0.27
Antidiarrheals	0.22
Home – Bronchodilators	0.19
Home - Anticoagulants and Thrombolytics	0.17
Antiarrhythmics	0.16
Anticonvulsants	0.15
Antivirals	0.15
Home – Antifungals	0.15
Home – Antiemetics	0.14
Laxatives	0.13
Antidepressants	0.12
Home - Sex Hormones (Female)	0.11
Sex Hormones (Female)	0.09
Analgesics	0.09
Muscle Relaxants	0.08
Home – Expectorant	0.07

Antineoplastics	0.07
Anticoagulants and Thrombolytics	0.07
Vitamins	0.06
Home – Immunosuppressives	0.06
Home – Antidiarrheals	0.04
Cough Suppressants	0.02
Home – Analgesics	0.02
Decongestants	0.01
Home – Decongestants	0.01
Home - Cough Suppressants	0.00
Antihistamines	0.00
Home - Sex Hormones (Male)	0.00
Home – Antineoplastics	0.00
Sex Hormones (Male)	0.00
Expectorant	0.00
Barbiturates	0.00
Home – Antidepressants	0.00
Home - Muscle Relaxants	0.00
Home – Corticosteroids	0.00

Table S1: CatBoost Global Feature Importance

Feature	Mean SHAP Impact	Direction
Pulse Rate	2.177	Positive (risk)
Resp Rate	1.643	Positive (risk)
BMI	-0.386	Negative (protective)
BP diastolic	-0.246	Negative (protective)
Home – Antibacterials	0.158	Positive (risk)
AGE	0.101	Positive (risk)
O2 Sat (percentage)	-0.087	Negative (protective)
BP systolic	0.062	Positive (risk)
Home - Anti-Inflammatories	-0.054	Negative (protective)
Antifungals	-0.052	Negative (protective)
Prior ED Discharge	-0.046	Negative (protective)
Antacids	0.030	Positive (risk)
Home – Antacids	0.024	Positive (risk)
Home – Antivirals	-0.023	Negative (protective)
Home – Diuretics	-0.021	Negative (protective)
Sleeping Drugs	0.019	Positive (risk)
Home – Vitamins	0.019	Positive (risk)
Antibacterials	0.017	Positive (risk)
Had_Catheter	-0.016	Negative (protective)
Height (cm)	0.015	Positive (risk)
SEX	-0.014	Negative (protective)
Home – Hormones	-0.012	Negative (protective)
Home - Sleeping Drugs	0.012	Positive (risk)
Home – Hypoglycemics	-0.009	Negative (protective)
Home – Antihistamines	-0.008	Negative (protective)
Diuretics	-0.008	Negative (protective)
Home - Beta-Blockers	-0.007	Negative (protective)
Home – Sedatives	-0.006	Negative (protective)
Antihypertensives	0.006	Positive (risk)
Beta-Blockers	-0.006	Negative (protective)

Hypoglycemics	-0.005	Negative (protective)
Home – Antipsychotics	-0.004	Negative (protective)
Home – Antihypertensives	-0.004	Negative (protective)
Antipyretics	0.004	Positive (risk)
Hormones	-0.004	Negative (protective)
Analgesics	-0.003	Negative (protective)
Anti-Inflammatories	-0.003	Negative (protective)
Immunosuppressives	-0.003	Negative (protective)
Sedatives	-0.003	Negative (protective)
Antiemetics	-0.002	Negative (protective)
Home – Expectorant	-0.002	Negative (protective)
Home - Anticoagulants and Thrombolytics	0.002	Positive (risk)
Vitamins	0.002	Positive (risk)
Antivirals	-0.002	Negative (protective)
Home – Laxatives	0.002	Positive (risk)
Antidiarrheals	0.002	Positive (risk)
Home - Antianxiety Drugs	-0.001	Negative (protective)
Home – Anticonvulsants	-0.001	Negative (protective)
Antipsychotics	0.001	Positive (risk)
Home – Antiarrhythmics	-0.001	Negative (protective)
Antidepressants	-0.001	Negative (protective)
Home – Immunosuppressives	-0.001	Negative (protective)
Home - Sex Hormones (Female)	0.001	Positive (risk)
Laxatives	0.001	Positive (risk)
Corticosteroids	-0.001	Negative (protective)
Antineoplastics	-0.001	Negative (protective)
Anticonvulsants	0.00	Positive (risk)
Cough Suppressants	0.00	Negative (protective)
Antianxiety Drugs	0.00	Positive (risk)
Bronchodilators	0.00	Positive (risk)
Home – Analgesics	0.00	Positive (risk)
Antiarrhythmics	0.00	Negative (protective)
Decongestants	0.00	Negative (protective)

Home – Decongestants	0.00	Negative (protective)
Muscle Relaxants	0.00	Negative (protective)
Home – Antiemetics	0.00	Negative (protective)
Home – Antifungals	0.00	Positive (risk)
Home – Bronchodilators	0.00	Positive (risk)
Sex Hormones (Female)	0.00	Positive (risk)
Anticoagulants and Thrombolytics	0.00	Positive (risk)
Home – Antidiarrheals	0.00	Negative (protective)
Expectorant	0.00	Positive (risk)
Home - Sex Hormones (Male)	0.00	Positive (risk)
Home – Antineoplastics	0.00	Positive (risk)
Home - Cough Suppressants	0.00	Positive (risk)
Antihistamines	0.00	Positive (risk)
Barbiturates	0.00	Positive (risk)
Home – Antidepressants	0.00	Positive (risk)
Home – Corticosteroids	0.00	Positive (risk)
Sex Hormones (Male)	0.00	Positive (risk)
Home - Muscle Relaxants	0.00	Positive (risk)

Table S2: All Features by Directional Mean SHAP Impact – CatBoost Model

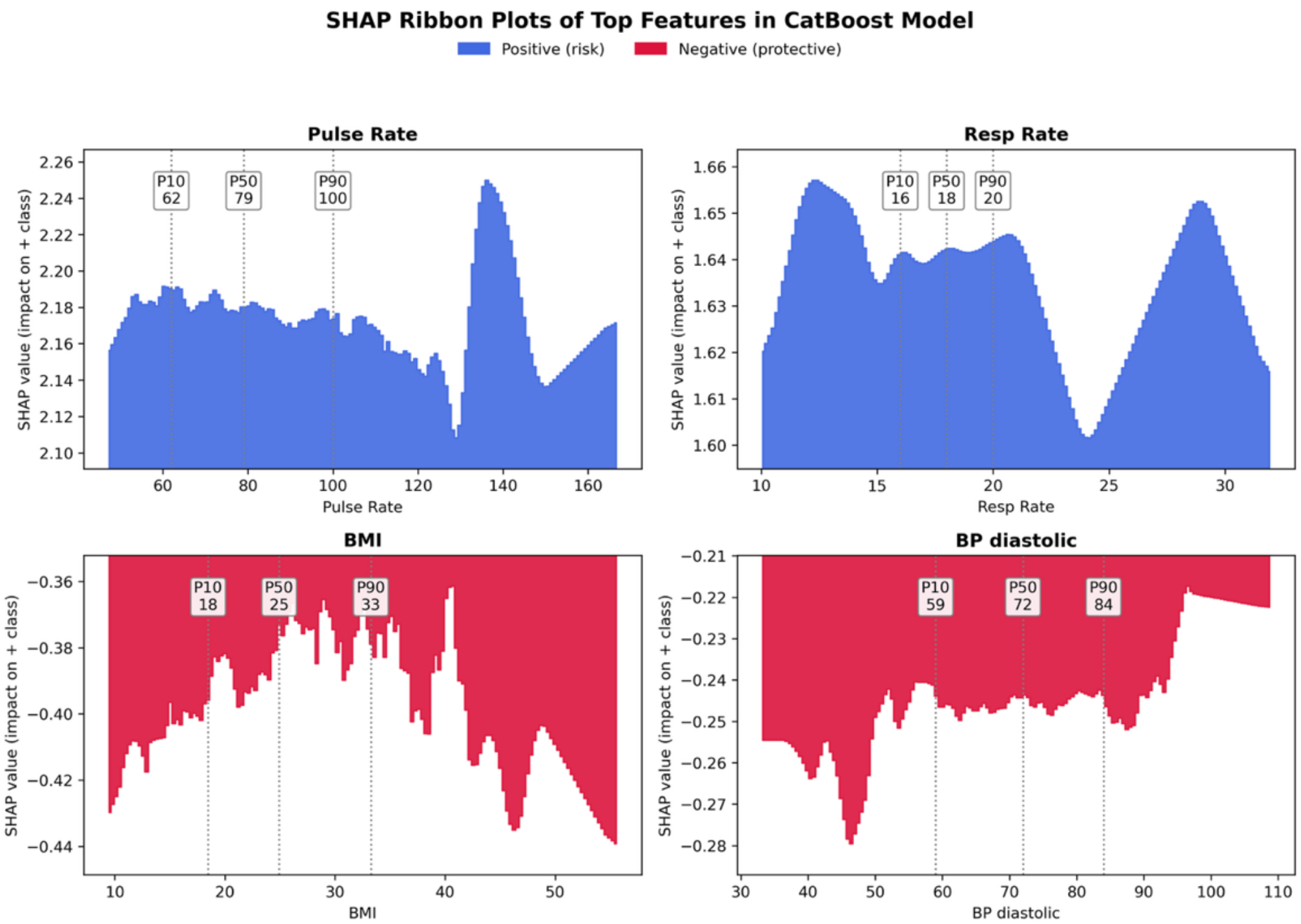

Figure S1: Binned SHAP ribbon plots for the top CatBoost features: Pulse Rate, Respiratory Rate, BMI, and diastolic blood pressure. The x-axis shows feature values and the y-axis shows SHAP values. Filled areas above zero (blue) indicate an average increase in predicted risk, and areas below zero (red) indicate a decrease. Curves are smoothed means of SHAP values within bins, with vertical dotted lines marking the 10th, 50th, and 90th percentiles of the evaluation data. Pulse rate and respiratory rate demonstrated consistently positive SHAP values, indicating that higher measurements were associated with increased predicted risk of deterioration. In contrast, BMI and diastolic blood pressure showed consistently negative SHAP values, indicating a protective association across their ranges. No feature demonstrated a reversal in direction of effect, supporting the stability and clinical plausibility of the model's learned relationships.

**Feature**	**Unit of change**	**OR (95% CI)**	P-value
Pulse Rate	per 1 SD	1.82 (95% CI 1.66–1.99)	$p < 0.0001$
Antifungals	1 vs 0	3.09 (95% CI 2.57–3.72)	$p < 0.0001$
Home – Antibacterials	1 vs 0	3.18 (95% CI 2.61–3.86)	$p < 0.0001$
Antibacterials	1 vs 0	3.03 (95% CI 2.49–3.68)	$p < 0.0001$
Prior ED Discharge	1 vs 0	3.53 (95% CI 2.81–4.44)	$p < 0.0001$
Resp Rate	per 1 SD	1.87 (95% CI 1.67–2.09)	$p < 0.0001$
Home – Diuretics	1 vs 0	2.66 (95% CI 2.21–3.19)	$p < 0.0001$
Home - Beta-Blockers	1 vs 0	2.31 (95% CI 1.93–2.78)	$p < 0.0001$
Home – Hypoglycemics	1 vs 0	2.31 (95% CI 1.92–2.78)	$p < 0.0001$
Hypoglycemics	1 vs 0	2.26 (95% CI 1.87–2.72)	$p < 0.0001$
Beta-Blockers	1 vs 0	2.20 (95% CI 1.83–2.65)	$p < 0.0001$
Home – Antifungals	1 vs 0	2.31 (95% CI 1.89–2.82)	$p < 0.0001$
O2 Sat (percentage)	per 1 SD	0.70 (95% CI 0.64–0.76)	$p < 0.0001$
Diuretics	1 vs 0	1.96 (95% CI 1.75–2.54)	$p < 0.0001$
Home – Antivirals	1 vs 0	2.28 (95% CI 1.85–2.80)	$p < 0.0001$
Home – Hormones	1 vs 0	2.09 (95% CI 1.67–2.41)	$p < 0.0001$
Immunosuppressives	1 vs 0	2.12 (95% CI 1.74–2.58)	$p < 0.0001$
Antacids	1 vs 0	2.73 (95% CI 2.07–3.61)	$p < 0.0001$
Home – Antacids	1 vs 0	2.39 (95% CI 1.84–3.09)	$p < 0.0001$
Hormones	1 vs 0	1.92 (95% CI 1.60–2.30)	$p < 0.0001$
BP diastolic	per 1 SD	0.82 (95% CI 0.75–0.90)	$p < 0.0001$
Bronchodilators	1 vs 0	1.86 (95% CI 1.53–2.26)	$p < 0.0001$
Home – Bronchodilators	1 vs 0	1.80 (95% CI 1.47–2.24)	$p < 0.0001$
Had_Catheter	1 vs 0	1.77 (95% CI 1.42–2.19)	$p < 0.0001$
Home – Antihistamines	1 vs 0	1.73 (95% CI 1.42–2.09)	$p < 0.0001$
Home – Sedatives	1 vs 0	7.04 (95% CI 3.15–15.74)	$p < 0.0001$
Sedatives	1 vs 0	7.04 (95% CI 3.15–15.74)	$p < 0.0001$
Vitamins	1 vs 0	3.52 (95% CI 2.09–5.91)	$p < 0.0001$
Home – Anticonvulsants	1 vs 0	1.55 (95% CI 1.29–1.86)	$p < 0.0001$
Anticonvulsants	1 vs 0	1.50 (95% CI 1.25–1.80)	$p < 0.0001$
Corticosteroids	1 vs 0	1.49 (95% CI 1.24–1.79)	$p < 0.0001$

Home – Corticosteroids	1 vs 0	1.49 (95% CI 1.24–1.79)	p < 0.0001
BP systolic	per 1 SD	0.82 (95% CI 0.75–0.90)	p < 0.0001
Antivirals	1 vs 0	1.84 (95% CI 1.39–2.44)	p < 0.0001
Expectorant	1 vs 0	2.46 (95% CI 1.61–3.76)	p < 0.0001
Home – Expectorant	1 vs 0	2.46 (95% CI 1.61–3.76)	p < 0.0001
BMI	per 1 SD	0.82 (95% CI 0.74–0.91)	p < 0.0001
Home – Antihypertensives	1 vs 0	1.59 (95% CI 1.26–2.01)	p < 0.001
Home – Antipsychotics	1 vs 0	1.51 (95% CI 1.22–1.88)	p < 0.001
Antihypertensives	1 vs 0	1.57 (95% CI 1.24–1.99)	p < 0.001
Antipsychotics	1 vs 0	1.50 (95% CI 1.21–1.87)	p < 0.001
Antiarrhythmics	1 vs 0	1.86 (95% CI 1.31–2.62)	p < 0.001
Antihistamines	1 vs 0	1.56 (95% CI 1.20–2.03)	p < 0.01
Home – Antiemetics	1 vs 0	1.35 (95% CI 1.13–1.62)	p < 0.01
Home – Antiarrhythmics	1 vs 0	1.75 (95% CI 1.22–2.53)	p < 0.01
SEX	1 vs 0	1.31 (95% CI 1.10–1.58)	p < 0.01
Antidiarrheals	1 vs 0	1.72 (95% CI 1.20–2.46)	p < 0.01
Home – Antidiarrheals	1 vs 0	1.85 (95% CI 1.38–2.49)	p < 0.01
Cough Suppressants	1 vs 0	1.89 (95% CI 1.18–2.90)	p < 0.01
Home - Cough Suppressants	1 vs 0	1.89 (95% CI 1.18–2.90)	p < 0.01
Antipyretics	1 vs 0	1.38 (95% CI 1.08–1.78)	p < 0.01
Sleeping Drugs	1 vs 0	1.53 (95% CI 1.05–1.52)	p = 0.02
Home - Sleeping Drugs	1 vs 0	1.53 (95% CI 1.05–1.52)	p = 0.02
Home - Muscle Relaxants	1 vs 0	1.30 (95% CI 1.05–1.62)	p = 0.02
Home – Immunosuppressives	1 vs 0	1.43 (95% CI 0.95–2.15)	p = 0.09
Muscle Relaxants	1 vs 0	1.20 (95% CI 0.96–1.50)	p = 0.11
Home - Sex Hormones (Female)	1 vs 0	0.53 (95% CI 0.24–1.18)	p = 0.12
Home - Anti-Inflammatories	1 vs 0	1.14 (95% CI 0.95–1.37)	p = 0.15
Home – Analgesics	1 vs 0	7.63 (95% CI 0.45–128.75)	p = 0.16
Sex Hormones (Female)	1 vs 0	0.60 (95% CI 0.28–1.29)	p = 0.19
Anti-Inflammatories	1 vs 0	1.14 (95% CI 0.94–1.39)	p = 0.19
Laxatives	1 vs 0	1.95 (95% CI 0.58–6.59)	p = 0.28
Home – Laxatives	1 vs 0	1.85 (95% CI 0.55–6.29)	p = 0.32
Height (cm)	per 1 SD	1.04 (95% CI 0.95–1.15)	p = 0.37

Home – Antineoplastics	1 vs 0	1.18 (95% CI 0.79–1.75)	p = 0.43
Antianxiety Drugs	1 vs 0	1.09 (95% CI 0.88–1.35)	p = 0.43
Antineoplastics	1 vs 0	1.11 (95% CI 0.73–1.70)	p = 0.62
AGE	per 1 SD	1.02 (95% CI 0.93–1.12)	p = 0.64
Home - Antianxiety Drugs	1 vs 0	1.05 (95% CI 0.84–1.31)	p = 0.66
Home - Sex Hormones (Male)	1 vs 0	1.08 (95% CI 0.75–1.55)	p = 0.67
Sex Hormones (Male)	1 vs 0	1.08 (95% CI 0.75–1.55)	p = 0.67
Antidepressants	1 vs 0	1.03 (95% CI 0.86–1.24)	p = 0.75
Barbiturates	1 vs 0	1.71 (95% CI 0.06–51.15)	p = 0.76
Analgesics	1 vs 0	1.03 (95% CI 0.85–1.25)	p = 0.78
Decongestants	1 vs 0	1.14 (95% CI 0.37–3.56)	p = 0.82
Home – Decongestants	1 vs 0	1.14 (95% CI 0.37–3.56)	p = 0.82
Home – Antidepressants	1 vs 0	1.00 (95% CI 0.83–1.21)	p = 0.96
Anticoagulants and Thrombolytics	1 vs 0	0.99 (95% CI 0.71–1.40)	p = 0.98
Home - Anticoagulants and Thrombolytics	1 vs 0	0.99 (95% CI 0.71–1.40)	p = 0.98

Table S3: Univariate Odds Ratio (All Features)

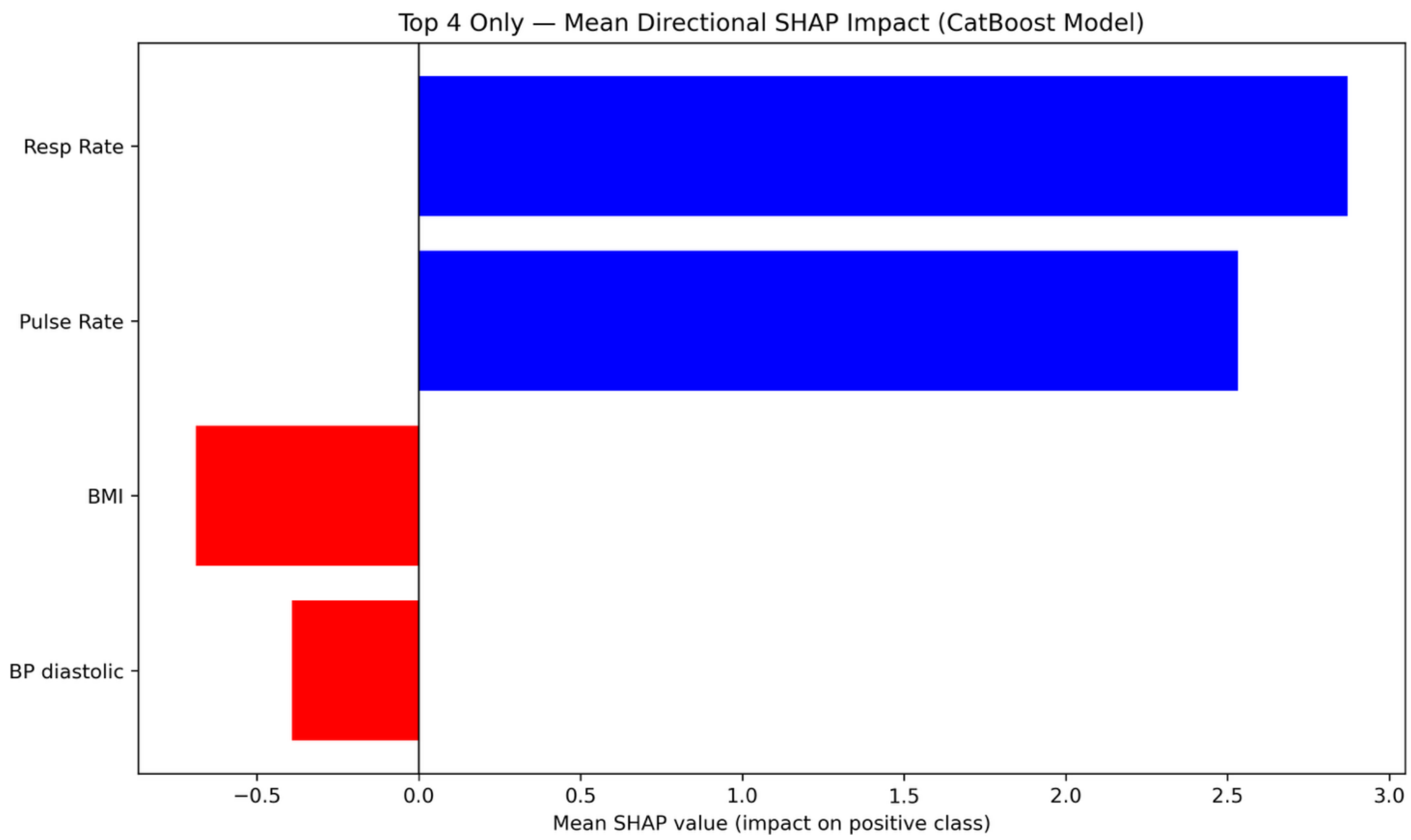

Figure S2: Mean Directional SHAP Impact (CatBoost Model) with only the top 4 contributing features

Feature	Mean SHAP	Direction
Resp Rate	2.8717	Positive (risk)
Pulse Rate	2.5319	Positive (risk)
BMI	-0.6895	Negative (protective)
BP diastolic	-0.3925	Negative (protective)

Table S4: Features by Directional Mean SHAP Impact – CatBoost Model including top 4 contributing features only

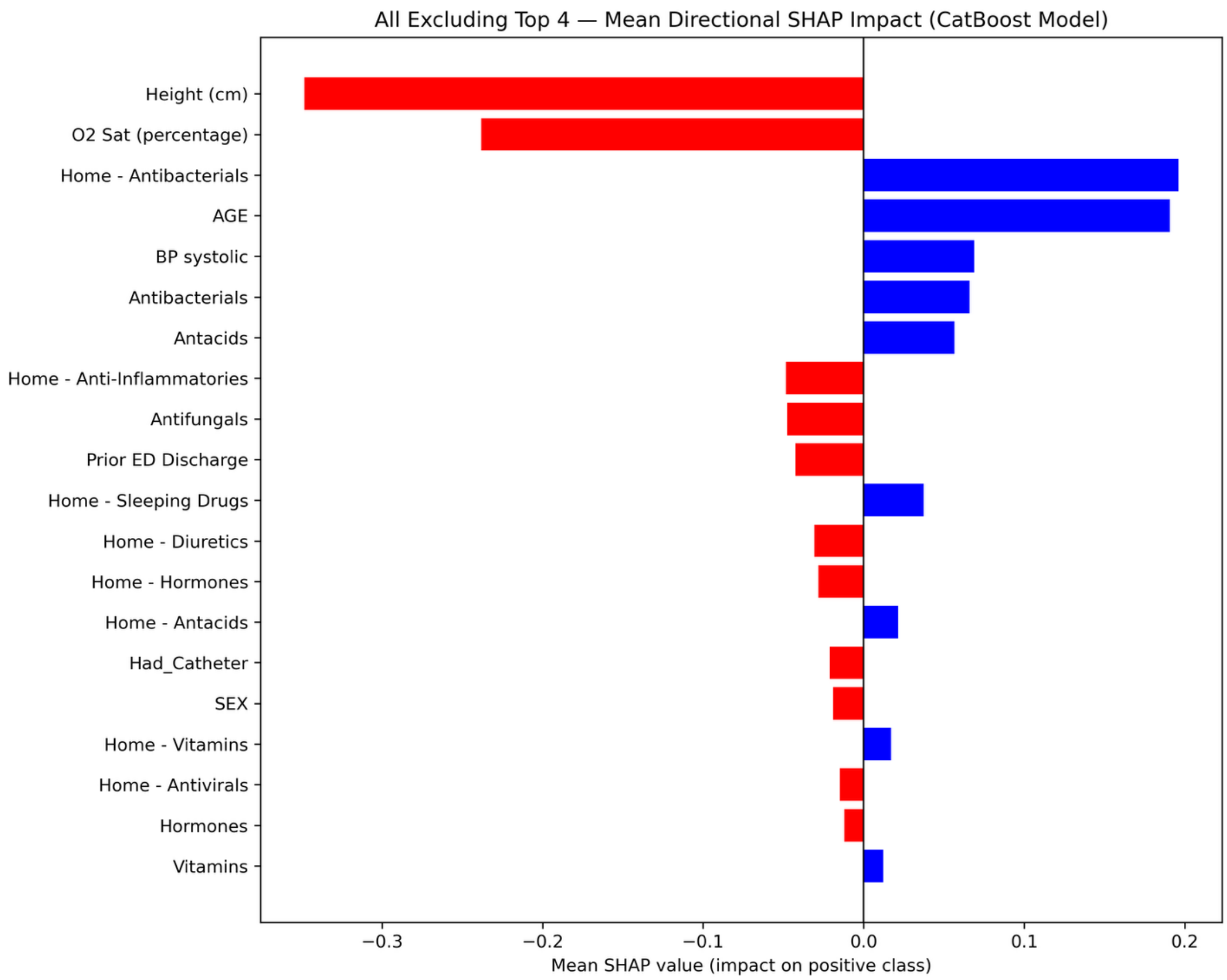

Figure S3: Mean Directional SHAP Impact (CatBoost Model) with all features other than the top 4 contributors.

Feature	Mean SHAP	Direction
Height (cm)	-0.3484	Negative (protective)
O2 Sat (percentage)	-0.2381	Negative (protective)
Home – Antibacterials	0.1961	Positive (risk)
AGE	0.1906	Positive (risk)
BP systolic	0.0689	Positive (risk)
Antibacterials	0.0658	Positive (risk)
Antacids	0.0567	Positive (risk)
Home - Anti-Inflammatories	-0.0484	Negative (protective)
Antifungals	-0.0477	Negative (protective)
Prior ED Discharge	0.0423	Negative (protective)
Home - Sleeping Drugs	0.0374	Positive (risk)
Home – Diuretics	-0.0308	Negative (protective)
Home – Hormones	-0.0289	Negative (protective)
Home – Antacids	0.0716	Positive (risk)
Had_Catheter	-0.0212	Negative (protective)
SEX	-0.0191	Negative (protective)
Home – Vitamins	0.0171	Positive (risk)
Home – Antivirals	-0.0148	Negative (protective)
Hormones	-0.0121	Negative (protective)
Vitamins	0.0121	Positive (risk)
Sleeping Drugs	-0.0117	Negative (protective)
Home – Hypoglycemics	-0.0113	Negative (protective)
Home - Beta Blockers	-0.0112	Negative (protective)
Immunosuppressives	-0.01	Negative (protective)
Antihistamines	-0.0098	Negative (protective)
Anti-Inflammatories	-0.0088	Negative (protective)
Beta Blockers	-0.0086	Negative (protective)
Home – Antihypertensives	0.0083	Positive (risk)
Diuretics	0.008	Positive (risk)
Home – Antidepressants	-0.0077	Negative (protective)
Home – Sedatives	-0.0073	Negative (protective)

Anticonvulsants	-0.0068	Negative (protective)
Hypoglycemics	-0.0064	Negative (protective)
Antiarrhythmics	-0.0054	Negative (protective)
Analgesics	-0.0047	Negative (protective)
Sedatives	-0.0043	Negative (protective)
Muscle Relaxants	-0.0041	Negative (protective)
Antipyretics	0.0041	Positive (risk)
Antianxiety Drugs	-0.0039	Negative (protective)
Home - Antianxiety Drugs	-0.0036	Negative (protective)
Home – Antiemetics	-0.0034	Negative (protective)
Sex Hormones (Male)	0.0033	Positive (risk)
Antiemetics	-0.0032	Negative (protective)
Cough Suppressants	0.0032	Positive (risk)
Home – Anticonvulsants	0.0032	Positive (risk)
Home – Antidiarrheals	-0.003	Negative (protective)
Antineoplastics	-0.003	Negative (protective)
Laxatives	0.0024	Positive (risk)
Home – Antihistamines	-0.0022	Negative (protective)
Antihypertensives	0.002	Positive (risk)
Antidepressants	0.0018	Positive (risk)
Anticoagulants and Thrombolytics	-0.0017	Negative (protective)
Home – Expectorant	-0.0017	Negative (protective)
Home – Laxatives	0.0015	Positive (risk)
Home - Cough Suppressants	-0.0014	Negative (protective)
Home – Antineoplastics	-0.0013	Negative (protective)
Corticosteroids	0.0012	Positive (risk)
Home – Immunosuppressives	-0.0011	Negative (protective)
Bronchodilators	-0.0011	Negative (protective)
Antivirals	-0.001	Negative (protective)
Antidiarrheals	-0.001	Negative (protective)
Home – Corticosteroids	-0.0009	Negative (protective)
Home – Bronchodilators	-0.0008	Negative (protective)
Home – Antiarrhythmics	0.0007	Positive (risk)

Home - Muscle Relaxants	-0.0006	Negative (protective)
Home – Antifungals	-0.0006	Negative (protective)
Home - Sex Hormones (Female)	0.0003	Positive (risk)
Home - Anticoagulants and Thrombolytics	0.0003	Positive (risk)
Home – Antipsychotics	0.0003	Positive (risk)
Home – Decongestants	0.0003	Positive (risk)
Home – Analgesics	0.0003	Positive (risk)
Sex Hormones (Female)	-0.0003	Negative (protective)
Antipsychotics	-0.0002	Negative (protective)
Expectorant	0.0002	Positive (risk)
Decongestants	-0.0001	Negative (protective)
Home - Sex Hormones (Male)	0	Negative (protective)
Barbiturates	0	Negative (protective)

Table S5: All Features by Directional Mean SHAP Impact – CatBoost Model excluding top 4 contributing features
